# Understanding the Potential Role of Sirtuin 2 on Aging: Consequences of SIRT2.3 Overexpression in Senescence

**DOI:** 10.3390/ijms22063107

**Published:** 2021-03-18

**Authors:** Noemi Sola-Sevilla, Ana Ricobaraza, Ruben Hernandez-Alcoceba, Maria S. Aymerich, Rosa M. Tordera, Elena Puerta

**Affiliations:** 1 Pharmacology and Toxicology Department, Faculty of Pharmacy, University of Navarra, Navarra Institute for Health Research (IdiSNA), 31008 Pamplona, Spain; nsola.4@unav.es (N.S.-S.); rtordera@unav.es (R.M.T.); 2 Gene Therapy Program CIMA, University of Navarra, Navarra Institute for Health Research (IdiSNA), 31008 Pamplona, Spain; aricobaraza@unav.es (A.R.); rubenh@unav.es (R.H.-A.); 3 Departamento de Bioquímica y Genética, Facultad de Ciencias, Universidad de Navarra, 31008 Pamplona, Spain; maymerich@unav.es; 4 Neuroscience Program CIMA, University of Navarra, Navarra Institute for Health Research (IdiSNA), 31008 Pamplona, Spain

**Keywords:** aging, sirtuin 2, senescence, Alzheimer’s disease, Parkinson’s disease, Huntington’s disease, neuroinflammation

## Abstract

Sirtuin 2 (SIRT2) has been associated to aging and age-related pathologies. Specifically, an age-dependent accumulation of isoform 3 of SIRT2 in the CNS has been demonstrated; however, no study has addressed the behavioral or molecular consequences that this could have on aging. In the present study, we have designed an adeno-associated virus vector (AAV-CAG-Sirt2.3-eGFP) for the overexpression of SIRT2.3 in the hippocampus of 2 month-old SAMR1 and SAMP8 mice. Our results show that the specific overexpression of this isoform does not induce significant behavioral or molecular effects at short or long term in the control strain. Only a tendency towards a worsening in the performance in acquisition phase of the Morris Water Maze was found in SAMP8 mice, together with a significant increase in the pro-inflammatory cytokine Il-1β. These results suggest that the age-related increase of SIRT2.3 found in the brain is not responsible for induction or prevention of senescence. Nevertheless, in combination with other risk factors, it could contribute to the progression of age-related processes. Understanding the specific role of SIRT2 on aging and the underlying molecular mechanisms is essential to design new and more successful therapies for the treatment of age-related diseases.

## 1. Introduction

Aging is the main risk factor for most diseases and conditions that reduce lifespan and quality of life [1]. It is a complex multifactorial biological process shared by all organisms with a gradual decline in normal physiological functions in a time-dependent manner. In addition, it increases the susceptibility to suffer from many diseases such as neurodegenerative diseases.

Currently, aging has gained importance due to the increase in world elderly population on account of the increase in human life expectancy and the reduction in death rates. By 2050, the number of people over 65 years old is expected to rise to 2.5 billion [2]; hence, understanding the molecular mechanisms involved in aging and identifying ways to improve quality of life are intriguing areas of biogerontology research.

Nine hallmarks have been identified in the process of aging: genomic instability, telomere attrition, loss of proteostasis, epigenetic alterations, deregulated nutrient sensing, mitochondrial dysfunction, cellular senescence, altered intercellular communication, and stem cell exhaustion [3]. Among them, epigenetic alterations represent a crucial mechanism behind the deteriorated cellular functions that can lead to changes in gene expression and are implicated in many age-related diseases [4]. One of the most studied is histone modification, which is characterized by post-translational changes at the *N*-terminal domains of histones. These modifications include methylation, ubiquitylation, phosphorylation, sumoylation, ribosylation, citrullination, and acetylation [4,5,6]. Histone acetylation, one of the most widely studied modification, is linked with longevity (for review see [7]). Histone acetyltransferases (HATs) and deacetylases (HDACs) are in charge of this process, and they regulate the balance between relaxed and repressed chromatin, which in turn regulates gene expression [8,9]. Based on their homology and phylogenetic relationship, four classes of HDACs have been defined in mammals: class I; class II which is divided into two subclasses—IIa and IIb; class III, also called sirtuins (SIRTs); and class IV. Although class I, II, and IV HDACs use zinc to catalyze hydrolysis of the acetylated lysines, SIRTs use the cofactor nicotinamide adenine dinucleotide (NAD^+^) [10]. SIRTs are involved in crucial cellular functions (for review, see [11,12,13,14,15,16]) and have been associated with aging, longevity, and neurodegenerative diseases [12,17].

The SIRT family is constituted by seven members (SIRT1-7), which are highly conserved from prokaryotes to eukaryotes [13]. All of them share the same catalytic domain formed by 275 amino acids, while they differ in the *N*-terminal and/or *C*-terminal sequences [18]. Regarding their functions, they catalyze post-translational modifications of proteins. All of them participate in the deacetylation of histone and non-histone substrates including transcriptional factors, enzymes, and other proteins [11,19]. However, some members, like SIRT4 and SIRT6, are also ADP-ribosyl transferases [19,20,21]. The SIRT members are ubiquitously distributed in all subcellular compartments. Specifically, SIRT1, SIRT6, and SIRT7 are mainly located in the nucleus, while SIRT3, SIRT4, and SIRT5 are mitochondrial enzymes [16,19,22]. Noteworthy, SIRT2 is the only member of the family found predominantly in the cytoplasm with the additional ability to translocate to the nucleus and also found in the mitochondria [23]. This supports the wide variety of substrates deacetylated by SIRT2 and its participation in multiple cellular processes such as senescence [24], cytoskeletal stabilization [25,26,27,28,29], myelin formation [30], oligodendrocyte differentiation (for review, see [31]), autophagy [23,25,29,32,33], and inflammation (for review, see [34]). Interestingly, among all seven sirtuins, SIRT2 has the strongest expression in the brain and is expressed in all brain cells including neurons and glial cells (oligodendrocytes, astrocytes, and microglia) [11]. Due to its specific characteristics, the relevance of SIRT2 has increased exponentially during the last years, and it has been proposed to have a central role in aging.

## 2. Results

### 2.1. SIRT2 Expression in Aging

Several studies have addressed differences in the expression of SIRT2 with aging, both in the periphery and in the central nervous system (CNS), leading to conflicting data and contradictory conclusions.

In the periphery, it has been demonstrated that *Sirt2* gene expression is significantly repressed with age in hematopoietic stem cells (HSCs) [35]. In agreement with this result, Luo et al. recently showed that there is a reduction in *Sirt2* expression together with an increase in mitochondrial stress in aged HSCs, which leads to an activation of NOD-, LRR-, and pyrin domain-containing protein 3 (NLRP3) inflammasome [36]. Noteworthy, similar effects have been described to happen in bone marrow derived macrophages [37]. In their study, He and co-workers recently demonstrated that *Sirt2* expression is decreased in macrophages isolated from old mice. Moreover, they show that NLRP3 deacetylation by SIRT2 regulates aging-associated inflammation and insulin resistance [37].

In agreement with these animal studies, it has been described that protein levels of SIRT2 decrease with age in human peripheral blood mononuclear cells, suggesting that it may have potential as a useful biomarker for monitoring health conditions and aging [38]. However, more recently a significant increase in *SIRT2* mRNA plasmatic levels has been reported in older subjects compared with healthy, young controls [39], and also an upregulation of SIRT2 as a marker of stress-induced premature senescence in multiple human cell lines [24]. Noteworthy, authors show that the induction of the senescence phenotype is accompanied not only by an increased expression of SIRT2 but also by a concomitant decline in the acetylation status of its downstream targets such as α-tubulin, nuclear factor κB (NF-kB), and H4K16 [24], three of the main substrates of SIRT2 [28,40,41,42,43,44,45].

**Figure 1:**
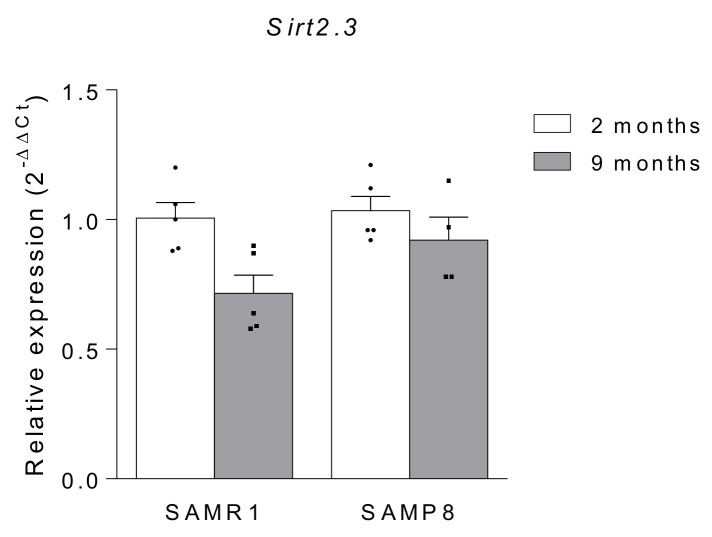
*Sirt2.3* mRNA expression in the hippocampus of 2 and 9 month-old SAMR1 and SAMP8 mice (*n* = 4–5 animals per group). *36b4* was used as internal control.

Regarding the CNS, the first study addressing the expression of SIRT2 with age was published by Maxwell and co-workers in 2011 [27]. In this study, the authors found that the three splice variants predicted for SIRT2, and referred as SIRT2.1 (sirtuin 2, isoform 1; calculated MW 43.2 kDa), SIRT2.2 (sirtuin 2, isoform 2; MW 39.5 kDa), and SIRT2.3 (sirtuin 2, isoform 3; MW 35.6 kDa), are expressed in the brain. Moreover, they showed, for the first time, a significant age-dependent accumulation of overall SIRT2 levels in the mouse brain and spinal cord, suggesting that SIRT2 levels are associated with aging in the CNS. Specifically, the authors demonstrated that isoform 3 is the one that shows the highest increase with age in C57BL/6J and B6CBA mouse models [27]. Accordingly, Diaz-Perdigon et al. have recently shown an increase in SIRT2 protein levels in the hippocampus of old senescence accelerated mouse-Prone 8 (SAMP8) and their corresponding controls senescence accelerated inbred mice (SAMR1) when compared with young animals [46]. Interestingly, we have analyzed *Sirt2.3* mRNA expression in 2 and 9 month-old SAMR1 and SAMP8 mice and found no significant differences among all four groups (Figure 1). This observation suggests that the increase in the protein levels observed by Maxwell et al. [27] and Diaz-Perdigon et al. [46] is due to a protein accumulation and not due to an increase in its synthesis.

Moreover, other studies have also assessed SIRT2 expression in the rat brain and have found different conclusions. While Kireev et al. found an age-related *Sirt2* decrease in the dentate gyrus of rats [47], Braidy et al. described an increase in mRNA and protein levels of SIRT2 only in the occipital lobe of these rodents [48]. Moreover, increased expression of *Sirt2* has been observed in the brain of 24 month-old rats and in a rat model of accelerated senescence induced by the administration of D-galactose [49].

**Figure 2:**
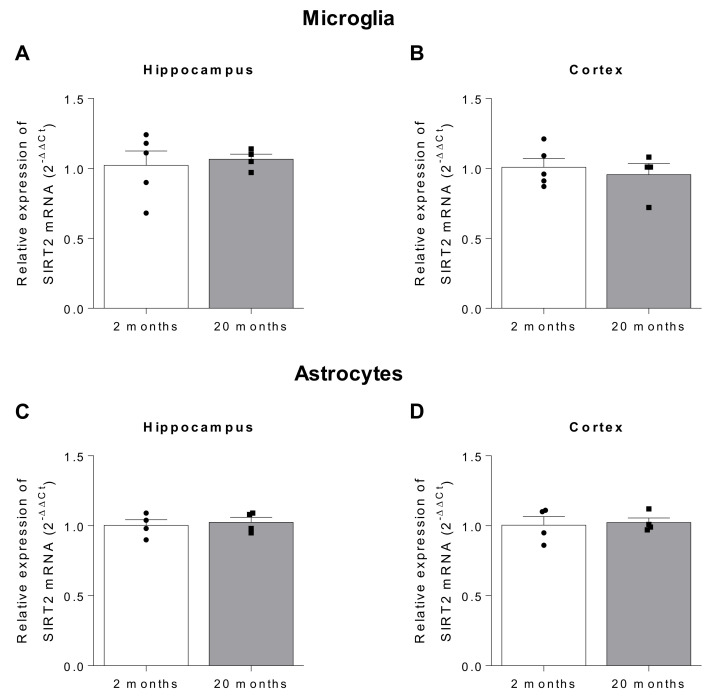
*Sirt2* expression in microglia (**A**,**B**) and astrocytes (**C**,**D**) of the hippocampus and cortex of young (2 month-old) and aged (20 month-old) C57BL/6J mice (*n* = 4–5 animals per group). *β-actin* was used as internal control.

Noteworthy, to date, none of these previous studies has described the expression of SIRT2 in each cell type of the CNS. In this context, we have analyzed the gene expression of SIRT2 in astrocytes and microglia of the cortex and hippocampus of 2 and 20 month-old C57BL/6J mice. As shown in Figure 2, no significant differences were observed in any of the regions studied, suggesting that the changes in SIRT2 expression described with age occur mainly at the neuronal level, as previously suggested [27].

Therefore, further studies are needed to fully understand the specific changes that occur with age in the expression of SIRT2 in different parts of the body and in different cell types, especially in neurons. In addition, it will be important to specify whether the changes detected are occurring at the protein or mRNA level. This would allow determining whether the changes observed are due to alterations in its synthesis or degradation. As it can be seen in Table 1, in a deep analysis of the different studies, the apparently contradictory results could be tissue-dependent or due to methodological differences to evaluate both mRNA and protein levels.

### 2.2. Potential Role of SIRT2 on Neurodegenerative Diseases

Despite the increasing studies trying to decipher the involvement of SIRT2 in aging, it has not been clarified the consequences that these changes in SIRT2 could have at the CNS level. In fact, there is not even a consensus whether they are a cause or consequence of age-related neuropathologies, an aspect that is crucial to determine the potential of SIRT2 as a therapeutic target for the treatment of neurodegenerative diseases.

#### 2.2.1. Is SIRT2 Beneficial for Age-Related Neurodegenerative Diseases?

Supporting the notion that increased SIRT2 in the brain is beneficial for aging, Singh et al. demonstrated in vitro that SIRT2 overexpression protected cells from rotenone or diquat induced cell death, whereas cell death was enhanced by SIRT2 enzymatic inhibition [50]. Moreover, they found higher enzymatic activity of SIRT2 in postmortem brain tissue from patients with different neurodegenerative diseases (Parkinson’s disease (PD), PD with dementia, dementia with Lewy bodies (DLB), and Alzheimer’s disease (AD)) compared to control samples [50]. The combination of these results leads the authors to suggest that elevated SIRT2 could be a compensatory mechanism to combat oxidative stress.

In fact, SIRT2 knockout (SIRT2^−/−^) has a detrimental effect on the normal function of the nervous system, which could be associated with age (for review, see [15]). Adult SIRT2^−/−^ mice show locomotor dysfunction due to axonal degeneration [51] and aberrant synaptic plasticity together with impaired learning and memory [52]. These effects are not present in young SIRT2^−/−^ mice [51], suggesting that SIRT2 is a key regulator of physiological aging.

In line with this hypothesis, it is postulated that SIRT2 could contribute to metabolism and longevity due to its link with caloric restriction. This dietary intervention is one of the most important strategies to improve brain health and retard aging [53]. Interestingly, Wang and coworkers reported an increased expression of SIRT2 in the white adipose tissue and kidneys of caloric-restricted mice [54]. They demonstrated that SIRT2-mediated deacetylation of Forkhead box O3 (FOXO3a) increases its binding to DNA, elevating the expression of FOXO target genes, (i.e., manganese superoxide dismutase) and decreasing cellular levels of reactive oxygen species. Nonetheless, under high oxidative stress conditions, SIRT2 could promote cell death in order to clear damaged cells. In this sense, they suggest that SIRT2 could be linked to caloric restriction and oxidative stress resistance, two crucial pathways in the control of the aging process [54]. Accordingly, North et al. described that BubR1, a key protein involved in longevity and aging, was increased by SIRT2 overexpression [55]. Interestingly, mice overexpressing BubR1 live longer than mice hypomorphic for this protein, which also show signs of accelerated aging. The authors demonstrated that as wild-type (WT) mice age, BubR1 levels decrease in many tissues due to a decline in NAD^+^ and the inability of SIRT2 to maintain lysine-668 of BubR1 deacetylated. Hence, increased BubR1 levels induced by the overexpression of SIRT2 enhanced the median lifespan in mice suggesting the potential of SIRT2 to delay aging and age-related diseases [55]. In addition, since caloric restriction is associated with increases in NAD^+^, they suggest the possibility that SIRT2-mediated increases in BubR1 could underlie some of the health benefits associated with this procedure [55].

#### 2.2.2. Is SIRT2 Detrimental for Age-Related Neurodegenerative Diseases?

Several authors have suggested that increased SIRT2 may be deleterious for neurons and promote neurodegeneration. First, it has been reported that SIRT2 interferes with stress-induced autophagy, autophagy-mediated degradation of protein aggregates under proteasome inhibition, and basal autophagy [32,33,56]. Noteworthy, in the context of neurodegenerative disorders, an emerging consensus is the view that an inadequate or defective autophagy promotes neuronal cell death, whereas induction of autophagy is a neuroprotective response [57]. In this context, it has been reported that the autophagy suppressor p53 is a major deacetylation substrate of SIRT2 [58]. An increase in p53 acetylation via SIRT2 inhibition reduces cytoplasmic p53 levels, thus blocking its inhibitory effect on autophagy [59], suggesting that SIRT2 inhibition may present a key pharmacological strategy in restoring autophagy function.

Second, an increase in SIRT2 levels has been described in different neuropsychiatric and neurodegenerative diseases including AD [29,39], PD [50,59,60,61], and Huntington’s disease (HD) [62], both in mouse models and human postmortem brain tissues (Table 2). Moreover, SIRT2 deletion or pharmacological inhibition has provided beneficial effects in different neurodegenerative diseases.

In this line, there is a general consensus on the beneficial effects of SIRT2 inhibition in PD. It was firstly demonstrated that inhibition of SIRT2 rescued α-synuclein toxicity in cellular models of PD [67,68]. More recently, it has been shown that α-synuclein acetylation is a key regulatory mechanism for α-synuclein aggregation and toxicity [69]. Interestingly, genetic SIRT2 deletion in vitro and in vivo increases α-synuclein acetylation and reduces its toxicity [69]. Moreover, it has been proposed that α-synuclein neurotoxicity is partially due to α-tubulin deacetylation by SIRT2 [25]. In this sense, SIRT2 inhibition would induce α-tubulin acetylation and would improve microtubule stability, increasing α-synuclein/tubulin binding, thus reducing α-synuclein toxicity. These results indicate that microtubules can be also a promising therapeutic target in the field of neurodegenerative disorders and that SIRT2 could play a key role in this process [25]. Noteworthy, SIRT2 deletion or inhibition also showed neuroprotective effects in MPTP-induced mouse models of PD [59,70].

Regarding HD, there are contradictory results concerning the role of SIRT2 in this disease. On the one hand, it has been proven that its genetic reduction or ablation had no effect on Huntingtin protein levels or disease progression in the genetic mouse model R6/2 [71]. On the other hand, several studies have demonstrated beneficial effects after SIRT2 inhibition in different cellular and animal models of HD [72,73,74,75], where transcriptional repression of cholesterol biosynthesis has been proposed as one of the underlying mechanisms [72].

In recent years, several studies have also confirmed that SIRT2 pharmacological inhibition could have therapeutic potential in AD. It has been demonstrated that SIRT2 inhibition in primary rat astrocytes challenged with amyloid-β 1-42 peptide reduces astrocyte activation as well as pro-inflammatory mediators’ production [76]. Moreover, the compound AK-1, a SIRT2 inhibitor, which was administered directly into the hippocampus, protected from neurodegeneration in a Tau transgenic mouse model without affecting neurofibrillary tangles [77]. In addition, other groups showed that SIRT2 inhibition improved cognitive performance in different AD transgenic mouse models via APP amyloidogenic processing [78,79] and Tau and microtubule stability modulation [78]. Accordingly, its inhibition or knock-out also improved autophagy and decreased typical AD cytoskeletal pathology [29], decreased Tau phosphorylation, and increased Tau/tubulin binding, improving microtubule dynamics [25]. In agreement with these studies, and supporting the detrimental effects of SIRT2 on aging, it has been recently shown that SIRT2 inhibition prevents cognitive deficits and neuroinflammation in the SAMP8 mouse model [46].

**Figure 3:**
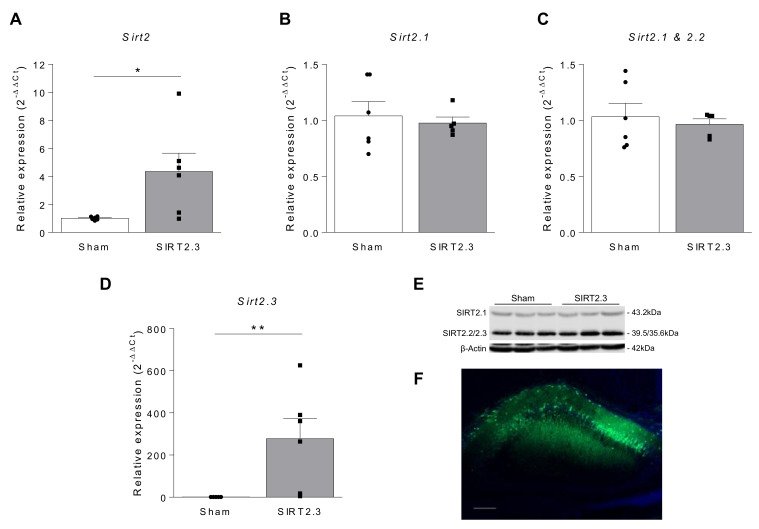
SIRT2 expression in SAMR1 mice treated with AAV-CAG-Sirt2.3-eGFP. Relative mRNA expression of total *Sirt2* (**A**) and the isoforms 1 (*Sirt2.1*) (**B**), 1 and 2 (**C**) and 3 (*Sirt2.3*) (**D**) of *Sirt2* in the hippocampus of SAMR1 mice 24 weeks after AAV injection (*n* = 6 animals per group). *Gapdh* was used as internal control. (**E**) Representative Western blot image of SIRT2 protein in the hippocampus of SAMR1 mice 24 weeks after AAV injection (*n* = 3 animals per group). β-actin was used as loading control. (**F**) Twenty-four weeks after the administration of AAV-CAG-Sirt2.3-eGFP, GFP (green) was detected in mice hippocampus. Scale bar = 200 μm. * *p* < 0.05, ** *p* < 0.001 vs. Sham group, Mann–Whitney U test.

In this context, we aimed to evaluate, for the first time, the behavioral and molecular consequences induced by the upregulation of the isoform 3 of SIRT2 (SIRT2.3), which, according to Maxwell and coworkers, shows a significant accumulation in the mouse brain and spinal cord with aging [27]. To this end, we have designed an adeno-associated virus vector (AAV-CAG-Sirt2.3-eGFP) for constitutive overexpression of SIRT2.3 and administered to 2 month-old mice through a bilateral stereotactic injection in the hippocampus region. In the first set of experiments, the AAV was administered to SAMR1 mice, in order to evaluate whether SIRT2.3 overexpression modified the aging process in a control strain. As shown in Figure 3A, a significant increase in *Sirt2* gene expression was detected in the hippocampus of vector-injected mice. As expected, this increase corresponded to the specific *Sirt2.3* isoform and neither to *Sirt2.1* nor to *Sirt2.2* (Figure 3B–D). Due to the extremely close migration of SIRT2.2 (39.5 kDa) and SIRT2.3 (35.6 kDa), and the overwhelming abundance of SIRT2.2 in the samples, these species are not easily resolved in mouse extracts. In this sense, the Western blot image of the microtubule-enriched fractions confirmed a stronger signal in the lower band (39.5–35.6 kDa) corresponding to SIRT2.2 and SIRT2.3 proteins in mice injected with AAV-CAG-Sirt2.3-eGFP (Figure 3E). The efficacy of the injected AAV was further confirmed by the analysis of GFP by immunofluorescence in brain slices (Figure 3F).

**Figure 4:**
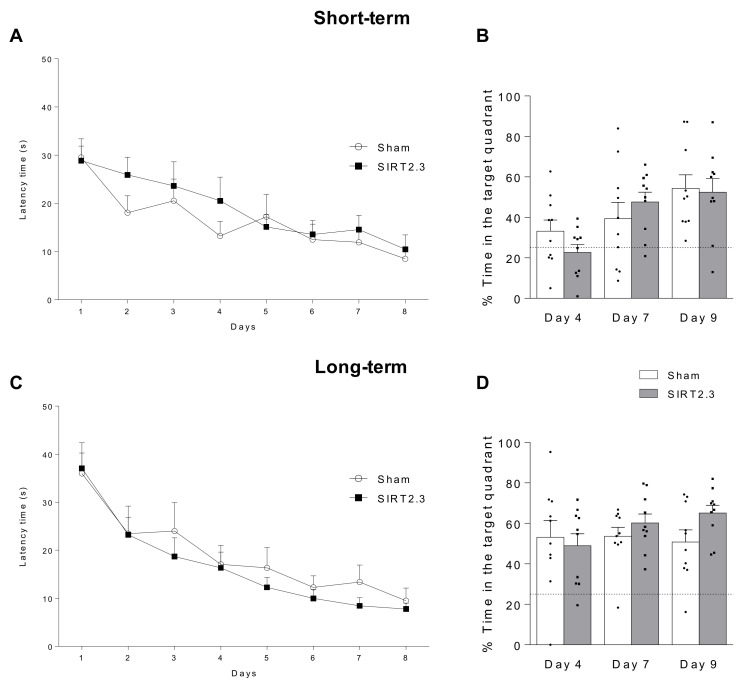
Behavioral consequences of SIRT2.3-overexpression in SAMR1 mice. Morris Water Maze (MWM) acquisition (**A**,**C**) and retention phases (**B**,**D**) at short and long-term (4 and 24 weeks after adeno-associated virus vector (AAV) injection, respectively) in SAMR1 mice (*n* = 10 animals per group).

**Figure 5:**
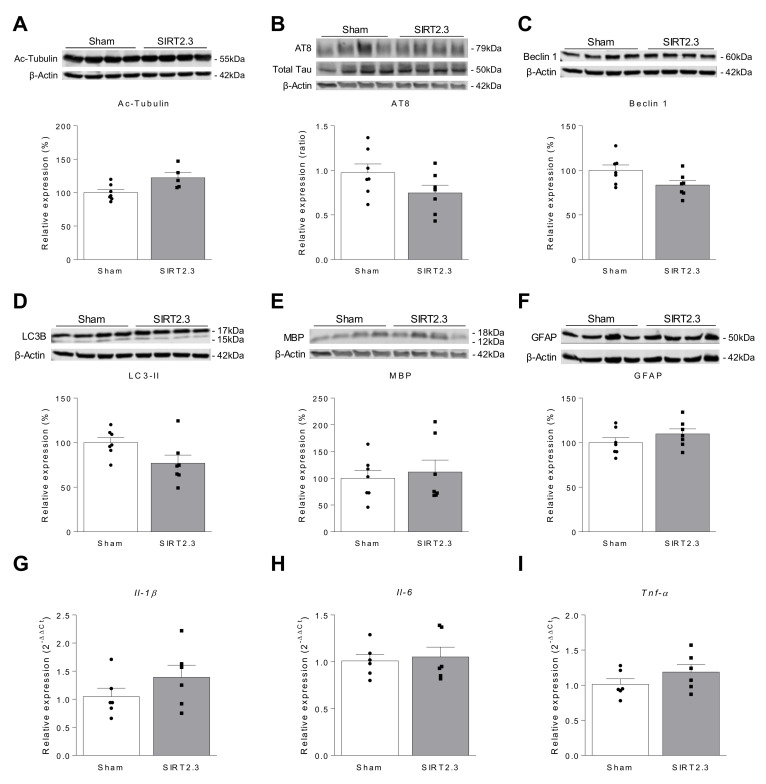
Molecular analysis of the hippocampus of SIRT2.3 overexpressing SAMR1 mice. Representative Western blot images and protein quantifications of acetylated alpha-tubulin (**A**), phospho-Tau (AT8 epitope) (**B**), autophagic markers Beclin 1 (**C**) and LC3-II (**D**), MBP (**E**) and GFAP (**F**). β-actin was used as loading control (*n* = 7 animals per group). No differences were found in gene expression of the pro-inflammatory cytokines *Il-1β* (**G**), *Il-6* (**H**), and *Tnf-α* (**I**). *Gapdh* was used as internal control (*n* = 6 animals per group).

Four and 24 weeks after the stereotactic injection, a hippocampal dependent memory test, the Morris Water Maze (MWM), was performed in order to evaluate the short and long term consequences of SIRT2.3 overexpression on the spatial memory. As shown in Figure 4, SIRT2.3 overexpression had no significant effects on the memory neither in the short (Figure 4A,B) nor in the long term (Figure 4C,D) in SAMR1 mice. Moreover, the molecular analysis of the hippocampus revealed no significant differences in the acetylation levels of alpha-tubulin (Figure 5A) nor in phospho-Tau expression (Figure 5B), and no significant differences were seen in the autophagic markers Beclin 1 and microtubule-associated protein light chain 3 type II (LC3-II) (Figure 5C,D). Taking into account that SIRT2 is highly expressed in oligodendrocytes [11], we next evaluated whether SIRT2.3 overexpression would have any effect on the myelin marker, myelin basic protein (MBP). However, as shown in Figure 5E, no differences were observed. Finally, we analyzed the effects of SIRT2.3 overexpression on neuroinflammation and found no differences in gene expression of the pro-inflammatory cytokines *Il-1β* (Figure 5G), *Il-6* (Figure 5H), and *Tnf-α* (Figure 5I) nor in the activated astrocytic marker, the glial fibrillary acid protein (GFAP) (Figure 5F).

**Figure 6:**
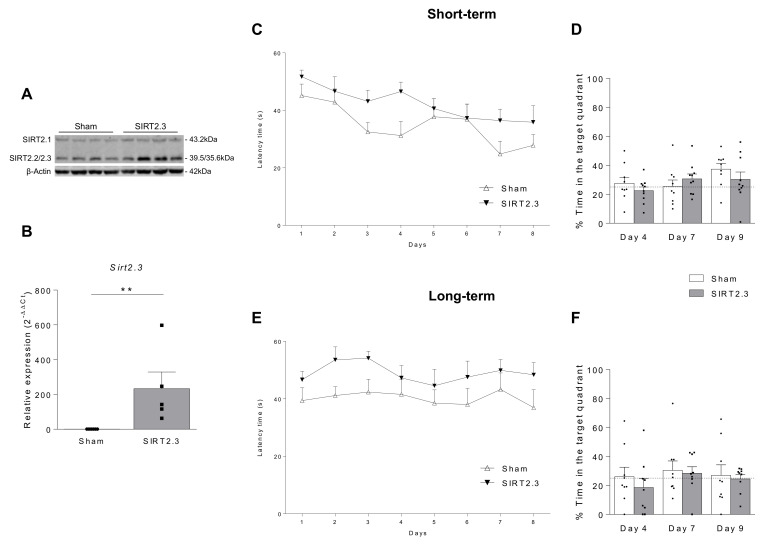
Behavioral consequences of SIRT2.3 overexpression in SAMP8 mice. (**A**) Representative Western blot image of SIRT2 protein (*n* = 4 animals per group; β-actin was used as loading control) and (**B**) relative mRNA expression of isoform 3 of *Sirt2* in the hippocampus of SAMP8 mice 24 weeks after AAV injection (*n* = 6 animals per group). *Gapdh* was used as internal control. MWM acquisition (**C**,**E**) and retention phases (**D**,**F**) at short and long-term (4 and 24 weeks after AAV injection, respectively) in SAMP8 mice (*n* = 10 animals per group). ** *p* < 0.001 vs. Sham group, Mann–Whitney U test.

**Figure 7:**
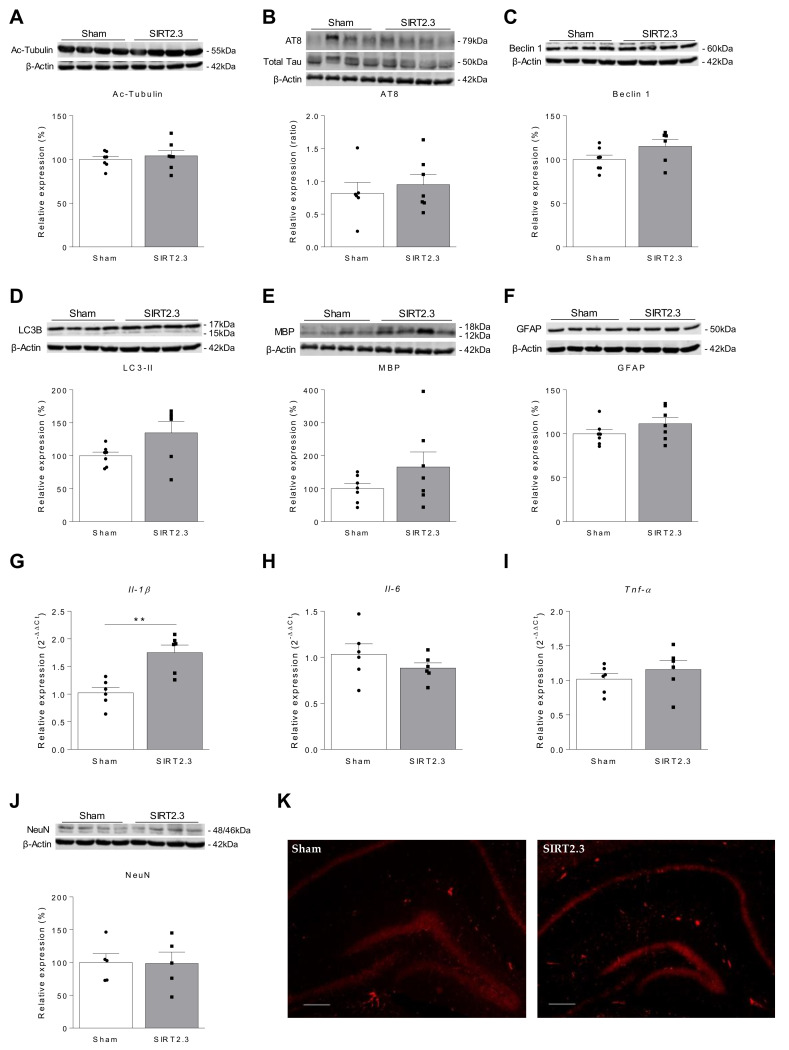
Molecular analysis of the hippocampus of SIRT2.3-overexpressing SAMP8 mice. Representative Western- blot images and protein quantifications of acetylated alpha-tubulin (**A**), phospho-Tau (AT8 epitope) (**B**), autophagic markers Beclin 1 (**C**) and LC3-II (**D**), MBP (**E**), and GFAP (**F**). β-actin was used as loading control (*n* = 7 animals per group). Note that a significant increase in the expression of *Il-1β* was found in SIRT2.3 overexpressing mice (**G**). No differences were found in *Il-6* (**H**) and *Tnf-α* (**I**). *Gapdh* was used as internal control (*n* = 6 animals per group). (**J**) Representative Western blot image and protein quantification of NeuN (*n* = 5 animals per group). β-actin was used as loading control. (**K**) Representative immunofluorescence images showing NeuN expression in the hippocampus of Sham and SIRT2.3-overexpressing SAMP8 mice. Scale bar = 200 μm ** *p* < 0.001 vs. Sham group, Mann–Whitney U test.

In this scenario, we next analyzed whether SIRT2.3 overexpression would have any beneficial or detrimental effects in a mouse model of accelerated aging, the SAMP8 strain, which has been proposed as a suitable rodent model for studying the molecular mechanisms underlying age-related cognitive impairment [80,81]. As it can be seen in Figure 6, in SAMP8 mice overexpressing SIRT2.3 (Figure 6A,B) it was observed a tendency towards impaired memory performance during the acquisition phase of the MWM performed at 4 and 24 weeks after the injection (Figure 6C,E, respectively). However, no significant differences were observed in the retention phase of the MWM neither in the short (Figure 6D) nor in the long term (Figure 6F). Noteworthy, since the SAMP8 model shows significant memory impairment in the MWM, it is very difficult to observe a worsening in this behavior. In this case, these results let us confirm, for the first time, that SIRT2.3 overexpression in the brain does not provide any beneficial effects on age-related cognitive decline. Moreover, in agreement with the results found in the control strain, the analysis of hippocampi showed no significant differences in acetylated alpha-tubulin (Figure 7A), phosphorylated Tau (Figure 7B), Beclin 1 (Figure 7C), LC3-II (Figure 7D), and MBP (Figure 7E). When pro-inflammatory cytokines were analyzed, a significant increase in *Il-1β* was found in mice overexpressing SIRT2.3 (Figure 7G). However, no significant differences were found in gene expression levels of the pro-inflammatory cytokines *Il-6* and *Tnf-α* (Figure 7H,I) nor in the astrogliosis marker GFAP (Figure 7F), ruling out the possibility that the overexpression of SIRT2.3 provoked a relevant neuroinflammatory effect in the brain. In addition, the analysis of the neuronal marker NeuN evidenced that the overexpression of SIRT2.3 was not inducing neuronal death or neurodegeneration since no significant differences were observed between Sham and AAV-injected mice (Figure 7J,K). These results indicate that, despite being a promising marker of aging [24,27], the isoform 3 of SIRT2 is not a cause of senescence nor a compensatory mechanism to combat aging; rather, it could be an effect linked to senescence-associated changes. Noteworthy, this concept is supported by the work of Anwar et al. in which the overexpression or knockdown of SIRT2 had no effect on stress-induced premature senescence in different cell lines, which led the authors to conclude that SIRT2 is not responsible for the induction or prevention of senescence [24]. However, further studies overexpressing and inhibiting specifically each SIRT2 isoform in different cell types are needed in order to fully understand the role of SIRT2 on aging.

### 2.3. Potential Role of SIRT2 on Inflammation

One of the principal characteristics of aging is the development of a mild pro-inflammatory state [82]. Inflammation is a natural process for the defense of pathogens and microorganisms, and the repair and maintenance of organs. However, it sometimes becomes prolonged leading to a state of chronic inflammation, the accumulation of damage and finally, pathology. This state, which correlates with aging and is called “inflammaging”, is a strong risk factor for the occurrence, progression, and complication of many chronic diseases such as cardiovascular and neurodegenerative diseases, and obesity [83]. Interestingly, a key role of SIRT2 in inflammation has also been proposed. However, its specific function is not clear, with different studies supporting that it can either prevent or promote it.

The deacetylation of the p65 subunit of NF-κB at lysine 310 by SIRT2 leads to a reduced expression of IL-1, IL-6, and TNF-α pro-inflammatory cytokines [42]. In line with these results, whole-body SIRT2 deletion or inhibition promoted inflammatory responses by increasing NF-κB acetylation and creating a pro-inflammatory milieu in a mouse model of colitis [84], in obese-septic mice [43], and in a mouse model of traumatic brain injury [44]. In animal models of collagen-induced arthritis, SIRT2 also inhibited the inflammation and improved physical impairment [85]. Remarkably, expression levels of *SIRT2* mRNA were dramatically decreased in extracellular plasma of rheumatoid arthritis patients compared with healthy controls [86]. In addition, Pais et al. found that SIRT2-deficient mice showed morphological changes in microglia and an increase in pro-inflammatory cytokines upon intracortical injection of lipopolysaccharide (LPS) [87]. Accordingly, it has been demonstrated that neuroinflammation and neuropathic pain can be alleviated by SIRT2 overexpression, while its inhibition aggravated both pathologies [45]. In line with this hypothesis, the cell penetrating fusion protein PEP-1-SIRT2 significantly blocked cytokine expression as well as NF-κB and mitogen-activated protein kinases (MAPK) activation under LPS stimulation [88]. It also protected against hydrogen peroxide-induced cell death and cytotoxicity in a mouse macrophage cell line [88]. Interestingly, supporting the anti-inflammatory role of SIRT2, two new substrates for SIRT2 have been recently identified: NLRP3 [36,37] and Heat-shock protein 90α (Hsp90α) [89]. During physiological aging, NLRP3 inflammasome is specifically activated in macrophages and contributes to the increased inflammatory milieu. In this context, SIRT2 overexpression in old macrophages reduced NLRP3 acetylation and activation, reversing aging-associated insulin resistance and neuroinflammation [37]. These results determine the SIRT2-NLRP3 pathway dysregulation as an origin of aging-associated chronic inflammation. Regarding Hsp90, it has been demonstrated that its deacetylation by SIRT2 leads to the dissociation from the glucocorticoid receptor (GR), and the subsequent translocation of GR to the nucleus, where it represses the expression of inflammatory cytokines. Accordingly, SIRT2 knock-down induced cytokine up-regulation [89]. Altogether, these studies point towards an anti-inflammatory role of SIRT2.

By contrast, suggesting a role of SIRT2 in promoting inflammation, several studies have shown in vitro that SIRT2 silencing or inhibition decreases nitric oxide and pro-inflammatory cytokines production induced by LPS administration in macrophages [90] and in the BV2 microglia cell line [91,92]. These results are supported by in vivo studies where pharmacological inhibition of SIRT2 activity resulted in a reduction of pro-inflammatory cytokine levels and survival improvement in a mouse model of lethal septic shock induced by a cecal-ligation-and-puncture [93]. Moreover, Ciarlo et al. have demonstrated that SIRT2 deficiency promotes bacterial phagocytosis by macrophages and protects from chronic staphylococcal infection [94]. In agreement, it has been suggested that SIRT2 is required for LPS-induced neuroinflammation and brain injury. The treatment with the selective SIRT2 inhibitor AGK2 decreases LPS-induced pro-inflammatory cytokines through regulation of the MAPK pathway [92,95]. Likewise, sevoflurane-induced neuroinflammation and microglial activation were decreased when rats were treated with the SIRT2 inhibitor AK7 [96].

To conclude, although apparently contradictory, all these studies indicate that SIRT2 may be an important modulator of inflammation. A clear picture will only be obtained when the underlying mechanisms and the relevance of different targets are unveiled in each specific context.

## 3. Discussion

Sirtuins represent a unique class of enzymes that not only regulate protein acetylation and metabolism, but also play prominent roles in promoting longevity, preventing disease, and improving cell survival. The in vivo and in vitro evidence presented above suggest that SIRT2 seems to be a crucial regulator of aging and age-related pathologies; nevertheless, controversial findings have been published on its specific role in these processes.

Several studies suggest that SIRT2 expression is altered in aging. However, whether this is as a consequence or a cause of senescence has not yet been clarified, and the full profile of molecular mechanisms remains controversial and incomplete. For example, some reports show that SIRT2 is downregulated with age in some cell types and animal models [35,36,38,47], while other studies show an upregulation [24,27,39,46,48].

There are also controversial findings regarding the interpretation and the molecular consequences of these changes in SIRT2 expression. While it has been suggested that SIRT2 accumulation in the brain with aging could be a compensatory mechanism to combat oxidative stress [50], other authors have provided evidence on the beneficial effects of SIRT2 inhibition in different age-related neurodegenerative diseases. In such a scenario, what is the role of SIRT2 in senescence?

Among all the published works, Maxwell et al. were the first to demonstrate the accumulation of isoform 3 of SIRT2 in the CNS with aging [27]. However, to date, no study has addressed the consequences that this could have on aging. Our results indicate that the specific overexpression of SIRT2.3 isoform in the mouse hippocampus does not induce significant behavioral or molecular effects in the short and long term. Our interpretation is that the age-related increase in SIRT2.3 found in the brain is not responsible for induction or prevention of senescence; instead, it is a protein abundantly expressed during the onset of senescent phenomenon. Noteworthy, no significant differences have been observed in the expression of well-known targets of SIRT2 such as acetylated α-tubulin (Figure 5A and Figure 7A and Supplementary Figure S1B), GluA1 (Supplementary Figure S2A,C), or the gene expression of the cholesterol transporter *Abca1* (Supplementary Figure S2B,D) in SIRT2.3 overexpressing samples, which questions the impact and functionality of this isoform. These results are in agreement with Maxwell et al. [27] who showed that the significant increase in SIRT2.3 found in the cortex of 18 month-old mice was associated with a modest increase in overall SIRT2 levels and only a slight reduction (not statistically significant) in acetylated α-tubulin. In any case, although no significant effects were obtained in the control strain with a normal aging (SAMR1 mice), a tendency towards a worsening in the behavior and a significant increase in the expression of *Il-1β* was found in the SAMP8 strain with a pathological phenotype. Therefore, we cannot rule out that although SIRT2.3 accumulation itself is not detrimental, in combination with other risk factors (oxidative stress, aggregation of toxic proteins, neuroinflammation, etc.), it could contribute to the progression of the disease. Thus, further studies are needed in order to clarify the effects of the overexpression of this specific isoform in other brain regions and other mouse models of neurodegenerative diseases.

Furthermore, SIRT2 may play a critical role in balancing the extent of an inflammatory reaction, which could be of great interest also in the context of aging and many inflammatory-related diseases. However, whether it plays a pro- or anti-inflammatory role is also controversial. Remarkably, the conclusions reached after the genetic deletion of SIRT2 do not always coincide with those where SIRT2 has been pharmacologically inhibited. It should be noted that, in addition to the possible impact of SIRT2 deletion during brain development, other factors may contribute to these conflicting conclusions, including compensatory mechanisms, the existence of different inflammatory scenarios for the same disease or the specificity and the dosage of the SIRT2 inhibitor. Thus, more complete in vivo studies by using conditional and cell-specific transgenic animals are needed to elucidate the mechanisms underlying the different functions of SIRT2.

In any case, regardless the putative changes in SIRT2 expression or activity with aging, the inhibition of SIRT2 appears to be another potential approach to treat neurodegeneration [7,15]. SIRT2 inhibitors can reduce neuroinflammation and cytotoxicity induced by toxins or mutant protein aggregation [69,92]. Furthermore, acetylation of α-tubulin also seems to be an important mechanism to exert neuroprotection [25,29,72]. Hence, these inhibitors show a promising strategy to target the main neuropathological hallmarks of different neurodegenerative diseases such as AD, PD, and HD. To sum up, it will be essential to understand the molecular mechanisms underlying the role of SIRT2 in each cell type both in healthy and pathological conditions, which could bring us closer to the identification of novel drug targets. These could be used to design new and more successful therapies and possibly even delay aging and treat age-related diseases.

## 4. Materials and Methods

### 4.1. Animals

Male SAMR1 and SAMP8 mice (2 months of age, bred from founders provided by Dr Pallas, University of Barcelona, Spain) were used for SIRT2.3 overexpression experiments. For microglia and astrocytes isolation, 2 and 20 month-old male C57BL/6J were used.

All mice were housed in groups in standard breeding cages and had access to food and water ad libitum. Temperature and humidity were constant (23 ± 1 °C and 55 ± 10%, respectively), and lights were maintained on a 12 h light/dark cycle (light-dark: 8:00AM–8:00PM). All rodents were weighed every week to control the loss of weight due to the procedures and behavioral tests. All the procedures followed in this study and animal husbandry were conducted according to the principles of laboratory animal care as detailed in the European Communities Council Directive (2013/53/EC) and were approved by the ethical committee of the University of Navarra. All efforts were made to minimize animal suffering and to reduce the number of animals used in the experiments.

### 4.2. Adeno-Associated Virus Production and Purification

A plasmid containing an expression cassette consisting of the ubiquitous CAG promoter (cytomegalovirus immediate-early enhancer and chicken β-actin promoter), an Internal Ribosomal Entry Site (IRES) and the enhanced GFP coding sequence was gently provided by Cristian Smerdou (CIMA, University of Navarra, Pamplona, Spain). The cassette is flanked by inverted terminal repeats (ITR) from AAV2. The coding sequence of SIRT2 isoform 3 (SIRT2.3) was obtained from GenScript Biotech (Leiden, The Netherlands). It was introduced in the CAG-IRES-eGFP cassette following the CAG promoter using NheI-HF and AscI restriction enzymes. The efficacy of the plasmid to successfully overexpress SIRT2.3 was further confirmed in the SH-SY5Y neuroblastoma cell line (Supplementary methods and Supplementary methods and Supplementary Figure S1A). To obtain AAV8 particles, the pro-viral plasmid was co-transfected with the helper pDP8 plasmid (pDP8.ape; Plasmid Factory, Bielefeld, Germany) in HEK293T cells using polyethylenimine (PEI; Sigma-Aldrich, St. Louis, MO, USA).

Seventy-two hours after transfection, cells were harvested by centrifugation and then were lysed in TMS 1X buffer (50 mM Tris-Cl, 150 mM NaCl, 2 mM MgCl_2_, 0.1% Triton X-100) and 3 cycles of freezing and thawing (−80 °C). After centrifugation to remove the debris, the supernatant was incubated with 0.001% PF-68 Pluronic F-68 non-ionic surfactant (Gibco, ThermoFisher Scientific, Waltham, MA, USA) at 4 °C until the purification. In the case of cell culture media, viral particles were precipitated with Polyethylene glycol 8000 (PEG; Sigma-Aldrich) at 4 °C for 72 h, followed by centrifugation at 1378× *g* for 15 min. The pellet was suspended in TMS 1X buffer and stored at −80 °C.

Viral particles were purified by ultracentrifugation at 350,000× *g* during 2.5 h in a 15–57% iodioxanol gradient using Ultraclear Centrifuge Tubes (Beckman Coulter, Brea, CA, USA). After centrifugation, the transparent layer was collected and concentrated by consecutive centrifugations using Amicon^®^ Ultra Centrifugal Filters (Merck Millipore, Burlington, MA, USA) and PBS supplemented with 5% sucrose and Pluronic F-68. Finally, the purified virus was concentrated in a total volume of 1 mL and stored at −80 °C. For viral quantitative analysis, DNA was extracted following the protocol from High Pure Viral Nucleic Acid Kit^®^ (Hoffmann-La Roche, Basel, Switzerland), and the eGFP gene was detected in CFX96 Touch™ Real-Time PCR Detection System (Bio-Rad, Hercules, CA, USA) using iQTM SYBR^®^ Green Supermix reagent (Bio-Rad).

### 4.3. AAV Injection

Adeno-associated viruses (AAV8-CAG-Sirt2.3-eGFP or AAV8 control virus, “Sham”) were administered to mice through a stereotactic injection in the hippocampus region. Mice were anaesthetized with a mixture of ketamine and xylacine (80 mg/kg and 10 mg/kg, respectively) and placed in the stereotactic equipment (Kopf Instruments, Tujunga, CA, USA). The skull was exposed, bregma pointed, and the virus was bilaterally injected with Hamilton syringe in the following coordinates: for SAMR1: −2 mm AP, ± 1.4 mm ML and −1.8 mm DV; for SAMP8: −1.9 mm AP, ±1.4 mm ML and −1.7 mm DV. A total of 10^9^ viral genomes (vg) were injected in each side (0.5 µL/min). After injection, the syringe was left in place for 2 min to allow complete diffusion of the virus solution, and then mice were sutured and observed until recovery from anesthesia. The behavioral tests were performed at 4 and 24 weeks after injection, and mice were sacrificed for biochemical analysis.

### 4.4. Behavioral Tests: Morris Water Maze

The Morris Water Maze (MWM) is a hippocampus-dependent learning task that is used to analyze the spatial memory and to assess the working and reference memory function. The water maze was a circular pool (diameter of 145 cm) filled with water (21–22 °C) and virtually divided into four equal quadrants (northeast, northwest, southeast, and southwest). Moreover, visual cues were placed in the room in order to guide the mice.

Firstly, mice underwent visible-platform training for 6 trials in one day, in which a platform was located in the southwest quadrant raised above the water with an object placed on top to facilitate its location (Habituation phase). In this phase, it was confirmed that all mice exhibited a normal swimming pattern and were able to reach the platform.

Next, learning capacity was tested (Acquisition phase). For this, a hidden platform (1 cm below the water surface) was placed in the northeast quadrant of the pool. The trial was finished when the animal reached the platform (escape latency) or after 60 s in the pool. After each trial, mice remained on the platform for 15 s. The test was conducted over 8 consecutive days (4 trials per day). To test memory retention, the platform was removed from the pool, and animals were allowed to swim for 60 s (Retention phase). Then, the percentage of time spent in the northeast quadrant was recorded. This trial was performed at the 4th, 7th, and 9th day of the test (last day). All trials were monitored by a video camera set above the center of the pool and connected to a video tracking system (Ethovision XT 5.0).

### 4.5. Microglia and Astrocytes Isolation

C57BL/6J mice were perfused transcardially with ice-cold phosphate-buffered saline (PBS) under xylazine/ketamine anesthesia. After perfusion, brains were removed, and the hippocampus and cortex were dissected out on ice. These regions were digested separately at 37 °C with rotation for 30 min with papain (2 mg/mL, Worthington Biochemical Corporation, Lakewood, NJ, USA) in Dulbecco’s PBS (Lonza, Basel, Switzerland), containing 50 μg/mL of DNase I (Sigma-Aldrich). Then, the tissue was mechanically dissociated and filtered through a 70 μm nylon cell strainer, followed by a centrifugation at 300× *g* in 25% Percoll gradient for 15 min.

Cell suspensions were incubated with CD11b MicroBeads at 1:10 dilution (Miltenyi Biotec, Bergisch Gladbach, Germany), and Microglial CD11b+ cells were separated on an autoMACS Pro Separator (Miltenyi Biotec), as previously described [97]. For astrocytes isolation, CD11b- cells were incubated with FcR Blocking Reagent (Miltenyi Biotec) for 10 min and then with ACSA-2 MicroBeads (1:10, Miltenyi Biotec,) for 15 min. Then, cellular suspensions were positively selected for ACSA-2 expression by autoMACS separator.

After separation, cells were pelleted and resuspended in the Homogenization Solution of Maxwell^®^ RSC simplyRNA Tissue Kit (Promega, Madison, WI, USA). Then, RNA extraction was performed following its protocol and was retro-transcribed into cDNA using the High-Capacity cDNA Reverse Transcription Kit (Applied Biosystems, Foster City, CA, USA).

### 4.6. RNA Extraction and Quantitative PCR

Total RNA was isolated from hippocampus samples using TRI Reagent^®^ (Sigma-Aldrich,) according to the manufacturer’s instructions. Two micrograms of RNA was retro-transcribed into cDNA using the High-Capacity cDNA Reverse Transcription Kit (Applied Biosystems), and quantitative real-time PCR was carried out on CFX384 Touch™ Real-Time PCR Detection System (Bio-Rad) using Taqman^®^ Universal PCR Master Mix (Applied Biosystems). Primers for *Sirt2*, Interleukin 1β (*Il-1β*), Interleukin 6 (*Il-6*), Tumor necrosis factor-alpha (*Tnf-α*), *Abca1*, *Gapdh*, and *β-actin* were used (Applied Biosystems).

For SIRT2 isoforms determination, quantitative real-time PCR was carried out using iQTM SYBR^®^ Green Supermix reagent (Bio-Rad). The primers used are detailed in the following table (Table 3). Due to the sequence of *Sirt2.2* gene, it is not possible to design a specific primer for its amplification; hence, *Sirt2.1* and *Sirt2.2* genes were detected together. The *36b4* gene expression was used as internal control.

For the quantification of gene expression, the double delta CT (∆∆CT) method was used where delta CT (∆CT) values represent normalized target genes levels with respect the internal control (*Gapdh*, *β-actin*, or *36b4*). The relative quantification of all targets was carried out using the comparative cycle threshold method, 2^−∆∆Ct^, where ∆∆Ct = (Ct target gene − Ct endogenous control) treated/(Ct target gene − Ct endogenous control) untreated.

### 4.7. Western Blot

For Western blot analysis, hippocampal tissues were sonicated in cold lysis buffer with protease inhibitors (0.2 M NaCl, 0.1 M HEPES, 10% glycerol, 200 mM NaF, 2 mM Na_4_P_2_O_7_, 5 mM EDTA, 1 mM EGTA, 2 mM DTT, 0.5 mM PMSF, 1 mM Na3VO4, 1 mM benzamidine, 10 mg/mL leupeptin, 400 U/mL aprotinin) and incubated on ice for 30 min. After centrifugation at 13,000 rpm for 20 min, the supernatant was collected. For SIRT2 expression, the pellet fractions were solubilized by addition of 1/10-volume TCE buffer (50 mM Tris-Cl at pH 7.0, 2% SDS, 10% glycerol and 1 mM DTT), followed by two rounds of boiling (100 °C, 5 min) and sonication (1 min) as previously described [27]. To measure total protein concentration, Bio-Rad protein assay was performed, following the manufacturer’s protocol (Bio-Rad).

Equal amounts of protein (30 µg) were separated by electrophoresis on a sodium dodecyl sulphate-polyacrylamide gel (7.5%) under reducing conditions and transferred onto a nitrocellulose membrane (Hybond-ECl; Amersham Bioscience, Amersham, UK) for 16 h. The trans-blots were blocked in TBS-Tween containing 5% powder milk for 1 h. Membranes were probed overnight at 4 °C with the following primary antibodies (all of them at 1:1000 dilution): rabbit polyclonal antibodies included anti-Beclin1 (Cell Signaling Technology, Danvers, MA, USA), anti-LC3B (Abcam, Cambridge, UK), anti-SIRT2 (Sigma-Aldrich), and anti-GluA1 (Sigma-Aldrich); rabbit monoclonal antibody anti-myelin basic protein (MBP) (Cell Signaling Technology); mouse monoclonal antibodies included anti acetylated-tubulin (clone 6-11B-1; Sigma-Aldrich), anti-AT8 (phospho-Tau Ser202, Thr205) (Thermo Fisher Scientific, Waltham, MA, USA), anti-glial fibrillary acidic protein (GFAP) (Cell Signaling Technology), anti-total Tau (Sigma-Aldrich) and anti-NeuN (clone A60; Merck). As internal control, mouse monoclonal anti-β-Actin was used (1:1000; Sigma-Aldrich).

The following day, membranes were incubated with goat polyclonal anti-rabbit and anti-mouse secondary antibodies (1:5000; Odyssey, LI-COR Biosciences, Lincoln, NE, USA) for 2 h at RT. Bands were visualized using Odyssey Infrared Imaging System (LI-COR Biosciences). Results were calculated as the optical density values of the control SAMR1 and SAMP8 mice.

### 4.8. Immunofluorescence

The brains of three mice per experimental group were histologically processed to confirm viral expression in the hippocampus. For this purpose, one brain hemisphere was postfixed for 24 h with paraformaldehyde 4% after dissection and conserved in sucrose 30% for 1 week. Serial brain slices (thickness: 30 μm) were cut with a freezing microtome and stored in cryoprotectant solution.

Free-floating slices, comprising the hippocampus, were washed 3 times in PBS and incubated in blocking solution (PBS containing 0.5% Triton X-100, 0.1% bovine serum albumin, and 2% normal donkey serum) for 2 h at RT. Sections were then incubated with rabbit polyclonal anti-GFP primary antibody (1:500; Thermo Fisher Scientific) or mouse monoclonal anti-NeuN primary antibody (1:500; clone A60; Merck) overnight at 4 °C. Next, slices were washed with PBS and incubated with the secondary antibody Alexa Fluor donkey anti-rabbit 488 or Alexa Fluor donkey anti-mouse 546 (1:200; Thermo Fisher Scientific) and Dapi for 2 h at RT, protected from light. Finally, sections were washed with PBS and mounted with Shandon™ Immu-Mount™ (Thermo Fisher Scientific).

To ensure comparable immunostaining, sections were processed together under identical conditions. Images were acquired with the AxioCam MR R3 camera and the program ZEN 2 blue edition (Zeiss Microscopy, Jena, Germany). Acquired fluorescence images were adjusted in parallel for brightness and contrast in ZEN 2 blue edition (Zeiss Microscopy). Sharpness was improved employing an unsharp mask filter (Radius (sigma): 1.0 px, and mask weight; 0.60).

### 4.9. Statistical Analysis

Behavioral tests were analyzed by repeated-measures two-way ANOVA followed by multiple comparisons with Tukey’s test. For *Sirt2.3* gene expression in 2 and 9 month-old SAMR1 and SAMP8 mice, Kruskal Wallis test was performed. Biochemical results analysis and *Sirt2* expression in isolated microglia and astrocytes in aging were analyzed by the nonparametric Mann–Whitney U test. Results were expressed as mean ± standard error of the mean (SEM), and differences among groups were considered statistically significant at *p* < 0.05. All the statistics were performed by GraphPad Prism software (San Diego, CA, USA).

## Figures and Tables

**Table 1:** SIRT2 expression in aging.

Author, Year	Model-Species		Sample	SIRT2 Expression		Reference
	Model	Ages Compared		Effect with Aging	Gene or Protein	
Chambers et al., 2007	Mouse C57BL/6	2 vs. 21 months old	Isolated mouse hematopoietic stem cells from bone marrow	Decrease	mRNA	[35]
Maxwell et al., 2011	Mouse (both sexes) (1) C57BL/6J (2) B6CBA	(1) 4 vs. 19 months old (2) 3 vs. 18 months old	(1) Spinal cord (2) Cortex	Increase	Protein	[27]
Luo et al., 2019	Mouse (both sexes) C57BL/6	3 vs. 24 months old	Isolated mouse hematopoietic stem cells from bone marrow	Decrease	mRNA	[36]
Diaz-Perdigon et al., 2020	Mouse (male) SAMR1 and SAMP8	2 vs. 9 months old	Hippocampus	Increase	Protein	[46]
He et al., 2020	Mouse (male) C57BL/6	3 vs. 24 months old	Isolated macrophages	Decrease	mRNA	[37]
Kireev et al., 2013	Rat (male) Wistar	2 vs. 22 months old	Dentate gyrus (brain)	Decrease	mRNA	[47]
Braidy et al., 2015	Rat (female) Wistar	3, 12 and 24 months old	Occipital lobe (brain)	Increase	mRNA and protein	[48]
Garg et al., 2017	Rat (male) Wistar	(1) 4 vs. 24 months old (2) young controls vs. D-galactose induced accelerated senescence rat model	(1) Whole brain (2) Whole brain	(1) Increase (2) Increase	mRNA	[49]
Yudoh et al., 2015	Human (both sexes)	20–58 years old vs. old subjects	Peripheral blood mononuclear cells	Decrease	Protein	[38]
Wongchitrat et al., 2018	Human (both sexes)	25–35 years old vs. old subjects (≥ 65 years)	Plasma (peripheral blood)	Increase	mRNA	[39]

**Table 2:** SIRT2 expression in neurodegenerative diseases.

Author, Year	Model-Species		Sample	SIRT2 Expression		Reference
	Model	Groups Compared		Expression in the Disease	Gene or Protein	
Yang et al., 2006	Human samples Mesial temporal lobe epilepsy	Control vs. patients	Hippocampus	Decrease	Protein	[63]
Erburu et al., 2015	(1) Mouse C57BL/6J (2) Postmortem samples depressed patients	(1) Control vs. Chronic social defeat stress model (2) Control vs. patients	(1) Prefrontal cortex (2) Prefrontal cortex	(1) Increase (2) Increase	mRNA	[64]
Guan et al., 2016	Mouse C57BL/6 MPTP model of PD	(1) 12 week old control vs. MPTP mice (2) 72 week old control vs. MPTP mice	Substantia nigra	(1) No changes (2) Increase	Protein	[60]
Guerreiro et al., 2017	Mouse (male) a-SynA53T model of PD	17 months old control vs. transgenic mice	Whole Brain	Decrease	mRNA	[65]
Silva et al., 2017	Postmortem human brains of AD	Control vs. AD patients	Temporal cortex	Increase	Protein	[29]
Singh et al., 2017	Postmortem human samples of PD, PDD, DLB, AD	Control vs. patients	Frontal and temporal cortex	(1) Increase in PD and DLB (2) Increase activity in all diseases	(1) Protein (2) Activity	[50]
Harrison et al., 2018	Postmortem human brains of PD	Control vs. PD patients	Substantia nigra pars compacta	No changes	mRNA	[66]
Sun et al., 2018	Mouse C57BL/6 MPTP model of PD	12 weeks old control vs. MPTP mice	Midbrain	Increase	Protein (no changes in mRNA)	[59]
Wongchitrat et al., 2018	Human samples of AD (≥65 years old)	Control vs. AD patients	Plasma	No changes	mRNA	[39]
Baldo et al., 2019	Postmortem human brains of HD	Control vs. HD patients	Striatum (brain)	Increase	mRNA	[62]
Chen et al., 2019	Human samples of PD	Control vs. HD patients	Peripheral blood leukocytes	Increase	mRNA	[61]

Abbreviations: AD: Alzheimer’s disease; DLB: Dementia with Lewy Bodies; HD: Huntington’s disease; PD: Parkinson’s disease; PDD: PD with dementia.

**Table 3:** Primers sequences used for SYBR Green qPCR analysis.

	Forward Primer (5′-3′)	Reverse Primer (5′-3′)
*Sirt2.1*	TCAGGATTCAGACTCGGACAC	TGTAGCGTGTCACTCCTTCG
*Sirt2.1&2.2*	AGCGAGCGCTGCCGCAAG	AAGATGGCCTCTGGGTAAGGAAGGTG
*Sirt2.3*	AGCCGGACCGCCGCAAGG	AAGATGGCCTCTGGGTAAGGAAGGTG
*Gapdh*	CCAAGGTCATCCATGACAAC	TGTCATACCAGGAAATGAGC
*36b4*	AACAATCTCCCCCTTCTCCTT	GAAGGCCTTGACCTTTTCAG

## Data Availability

The data presented in this study are available on request from the corresponding author.

## Supplementary Materials

The following are available online at https://www.mdpi.com/1422-0067/22/6/3107/s1, Supplementary Methods; Supplementary Figures S1 and S2.

## Abbreviations

AAV: Adeno-associated virus; AD: Alzheimer’s disease; CNS: Central nervous system; DLB: Dementia with Lewy Bodies; FOXO3: Forkhead box O3; GFAP: glial fibrillary acid protein; GR: Glucocorticoid receptor; HAT: Histone acetyltransferase; HD: Huntington’s disease; HDAC: Histone deacetylase; HSC: Hematopoietic stem cells; Hsp90: Heat-shock protein 90; LC3-II: microtubule-associated protein light chain 3 type II; LPS: Lipopolysaccharide; MAPK: Mitogen-activated protein kinases; MBP: Myelin basic protein; MWM: Morris Water Maze; NAD^+^: Nicotinamide adenine dinucleotide; NLRP3: NOD-, LRR-, and pyrin domain-containing protein 3; NF-κB: Nuclear factor κB; PD: Parkinson’s disease; PDD: Parkinson’s disease with dementia; SAMP8: Senescence Accelerated Mouse-Prone 8; SAMR1: senescence accelerated inbred mice; SIRT: Sirtuin; SIRT2.1: Isoform 1 of SIRT2; SIRT2.2: Isoform 2 of SIRT2; SIRT2.3: Isoform 3 of SIRT2; SIRT2^−/−^: SIRT2 knockout mice; WT: Wild-type.
