# Supplementary material for: Understanding the Potential Role of Sirtuin 2 on Aging: Consequences of SIRT2.3 Overexpression in Senescence
Source: Int J Mol Sci. 2021 Mar 18;22(6):3107. doi: 10.3390/ijms22063107 (PMC8003096; doi:10.3390/ijms22063107)
Supplement: Supplementary file 1 [file ijms-22-03107-s001.pdf]

Supplementary Data

Supplementary Methods

Cell culture

SH-SY5Y human neuroblastoma cells were obtained from American Type Culture Collection (CRL-2266™, ATCC, VA, USA) and cultured according to standards procedures. SH-SY5Y cells were maintained in Dulbecco's Modified Eagle's Medium (DMEM) supplemented with GlutaMAX™, 10% fetal bovine serum (FBS), 1% MEM Non-essential Amino Acid Solution (100x) (BioWhittaker-Lonza, Basel, Switzerland), 10,000 U/ml penicillin and 10 mg/ml streptomycin (Invitrogen, Thermo Fisher Scientific, CA, USA). Cells were grown at 37ºC in a humidified atmosphere of 92% air / 8% CO_2.

Plasmids transfection

pAAV-CAG-Sirt2.3-eGFP and control plasmid were transfected using Lipofectamine® 2000 Reagent (Invitrogen, Thermo Fisher Scientific, CA, USA). Firstly, cells were seeded in p60 dishes (2.5 million of cells per dish) and twenty-four hours after plating, transfection was performed. For each p60 dish, 15 µg of plasmid were prepared in OPTIMEM® and incubated for 5 min at room temperature. Separately, Lipofectamine® and OPTIMEM® were incubated for 5 min at room temperature. Both mixes were merged and incubated for 5 min at room temperature. The final solution was added to each dish and cells were incubated at 37 ºC. After six hours, the medium was removed and replaced by fresh medium for additional forty-eight hours. GFP was visualized with fluorescence microscope and cells were collected and stored at -80ºC for Western-Blot analysis following the same steps as in *4. Materials and Methods: Western-Blot* section.

Supplementary Figures

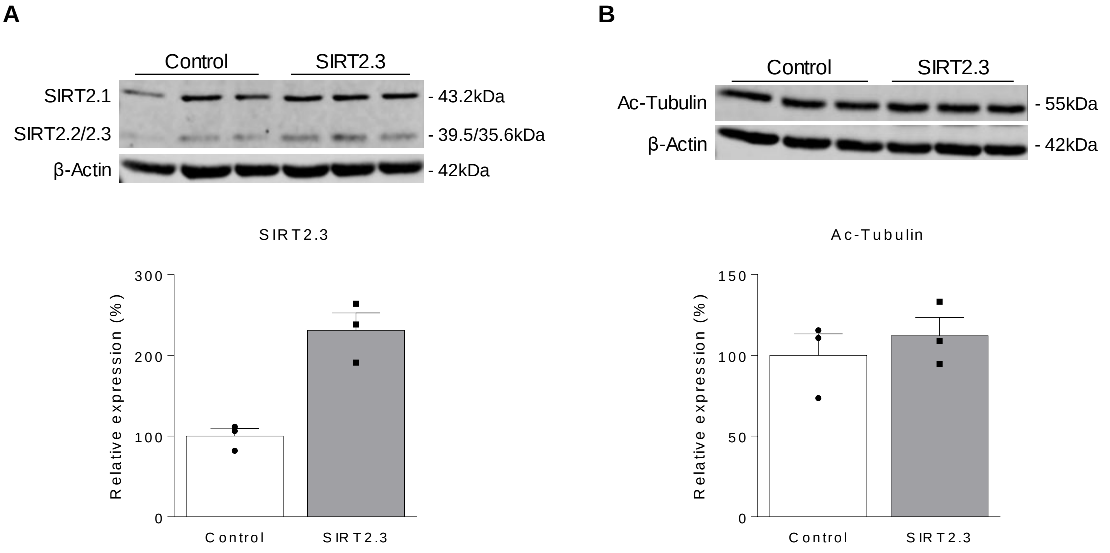

Supplementary Figure S1. Representative Western-Blot images and protein quantifications of SIRT2 (A) and acetylated alpha-tubulin (B) of SH-SY5Y transfected cells. β-actin was used as loading control.

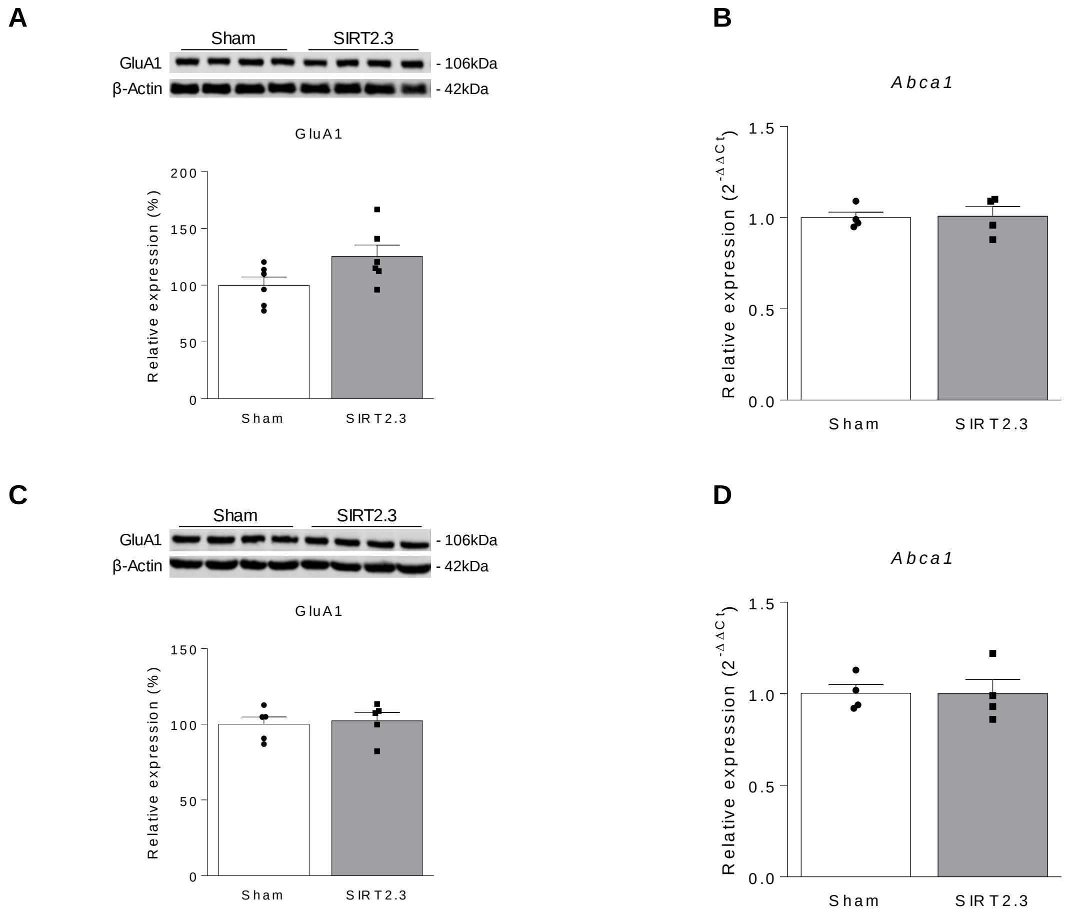

Supplementary Figure S2. Representative Western-Blot images and protein quantifications of hippocampal GluA1 (A, C), and *Abca1* gene expression (B, D) of SAMR1 (A, B) and SAMP8 mice (C, D). β-actin was used as loading control (n=5-6 animals per group) and *Gapdh* as internal control for qPCR analysis (n=4 animals per group).
